# Unique Oral Microbiota Signatures and Neurocognitive Function in Adolescents with and without Perinatally Acquired HIV

**DOI:** 10.21203/rs.3.rs-10920507/v1

**Published:** 2026-09-18

**Authors:** Modupe O. COKER, Anil KUMAR, Oluwaseun PETER, Ibukun J. ABULUDE, Qilei SHENG, Paul AKHIGBE, Esosa OSAGIE, Quindy Pan, Ahmad T. ALIYU, Jia LIU, Nosakhare L. IDEMUDIA, Ozoemene OBUEKWE, Stephanie SHIAU

**Affiliations:** University of Pennsylvania; University of Pennsylvania; Institute of Human Virology; University of Pennsylvania; Yale University; Institute of Human Virology; Institute of Human Virology; University of Pennsylvania; Institute of Human Virology; Emory University; University of Benin Teaching Hospital; University of Benin Teaching Hospital; Rutgers, The State University of New Jersey

**Keywords:** HIV, Cognition, Oral Health, Global Health, Microbiota, Cross-sectional

## Abstract

Adolescents with perinatally acquired HIV (PHIV) on antiretroviral therapy (ART) face early neurocognitive and oral disease, yet links between the oral microbiome and cognition remain underexplored. We analyzed baseline data from 53 PHIV and 47 age-, sex-, and socioeconomically matched HIV-unexposed-uninfected (HUU) adolescents (10–13 years) in Nigeria. Cognition was assessed with NeuroScreen and the NIH Toolbox. Oral rinse shotgun metagenomes were profiled for microbial diversity, community structure, and species-level differential abundance using five complementary methods stratified by HIV serostatus. Taxa were considered robust when concordant across at least two methods. Diversity and community structure did not significantly differ by HIV (Bray-Curtis PERMANOVA R^2^ = 0.017, p = 0.11) or across cognitive gradients, however at the species level, reproducible taxon-level signals emerged. In PHIV, *Leptotrichia*, *Tannerella*, *Actinomyces*, and *Prevotella* were associated with higher performance. In HUU, *Neisseria sp.* was associated with lower-cognitive performance across analyses in the HUU groups. *Streptococcus* associations differed in direction by HIV stratum. In this cross-sectional analyses, distinct oral taxa are associated with neurocognitive health in an HIV-context-dependent manner, identifying candidate markers of the oral-brain axis. Longitudinal, multi-omic studies are needed to test directionality and mechanism of action as candidate intervention targets.

## INTRODUCTION

Perinatally acquired HIV (PHIV) remains a significant public health concern in sub-Saharan Africa (SSA), which is home to over 80% of the world’s children living with HIV^1^. Despite advances in antiretroviral therapy (ART), children/adolescents with PHIV in SSA face a high risk of cognitive impairment that affects working memory, executive function, and processing speed, domains critical for educational attainment and independent functioning^2-4^. Meta-analyses demonstrate that children with PHIV score approximately 0.5 standard deviations lower on cognitive assessments compared to their uninfected peers, with deficits persisting despite early ART initiation and sustained viral suppression^5,6^. These impairments likely result from multiple interacting mechanisms such as chronic immune activation, delayed ART initiation, irreversible central nervous system injury before treatment, and socio-environmental stressors, collectively hindering developmental trajectories and quality of life^7^. These outcomes underscore the urgent need for integrated interventions that can identify at-risk populations and target modifiable biological contributors to cognitive impairment beyond traditional virologic control ultimately preserving neurodevelopment and other systemic sequalae in this growing population^8^.

Emerging evidence implicates the oral microbiome as a potential contributor to systemic inflammation and neurocognitive outcomes in people living with HIV (PWH)^9^. HIV infection and ART disrupt the oral microbial ecosystem, characterized by increased bacterial richness, altered community composition, and a shift toward potential pathogenic taxa^10-13^. The magnitude and direction of these shifts have been inconsistent across populations, and are often modest in children and adolescents on ART^14,15^. Where present, the dysbiosis drives chronic inflammation and systemic immune activation that may contribute to neurocognitive impairment^16^. Consistently with this, alterations in oral microbial diversity and composition have been associated with neurocognitive and neuropsychiatric conditions such as Alzheimer’s disease, dementia, and depression in adult populations^17-19^, and periodontal pathogens such as *Porphyromonas gingivalis* are associated with neurodegeneration and cognitive decline through systemic dissemination of inflammatory mediators and bacterial components^20^. Notably, these associations are most consistently detected at the level of specific taxa, suggesting that targeting the oral microbiome-brain axis may offer a modifiable therapeutic approach for HIV-associated neurocognitive disorders (HAND)^21^.

Despite increasing evidence linking oral microbiota to cognitive health in older adults^22^, little is known about these relationships in adolescents living with HIV, particularly in SSA, where the disease burden is greatest. This represents an important knowledge gap because adolescence is a critical window of neurodevelopmental vulnerability. Understanding whether and how HIV-associated oral dysbiosis contributes to cognitive impairment in adolescents could reveal novel biomarkers for early detection and open new therapeutic avenues to prevent or mitigate cognitive decline in this vulnerable population.

To address this gap, we characterized the oral microbial diversity and taxonomic composition in PHIV and HIV-unexposed-uninfected (HUU) adolescents and identified oral taxa that were differentially abundant by neurocognitive performance within each group. To our knowledge, this is the first study to examine associations between the oral microbiome and neurocognition in PHIV in SSA. By applying multiple complementary differential abundance methods, we aim to establish reproducible taxon-level associations that can inform future investigation of the oral-brain axis in this population.

## RESULTS

A total of 100 adolescents were enrolled, comprising 53 PHIV (53%) and 47 HUU (47%). The groups were well matched on age (mean 10.5 vs. 10.3 years, p = 0.20) and biological sex (38% vs. 43% male, p = 0.68) and were comparable in oral health (oral hygiene index simplified (OHIS) and decayed, missing, filled teeth (DMFT) scores similar between groups). PHIV showed a non-significant trend toward lower body mass index (BMI) (14.75 vs. 15.39 kg/m^2^, p = 0.07) and differed in mode of delivery, with a higher proportion of vaginal births (89% vs. 70%, p = 0.04). As expected, immunologic markers differed: mean CD4 count was 574 (SD 219) cells/μL in PHIV vs. 934 (SD 312) in HUU (p < 0.001), and mean CD8 count was 745 (SD 483) vs. 387 (SD 118) cells/μL (p < 0.001). Among PHIV, 28% (15/53) had a detectable viral load and the remainder were undetectable (Table 1). The cognitive scores were summarized stratified by HIV status under Table 2.

**Figure 1:**
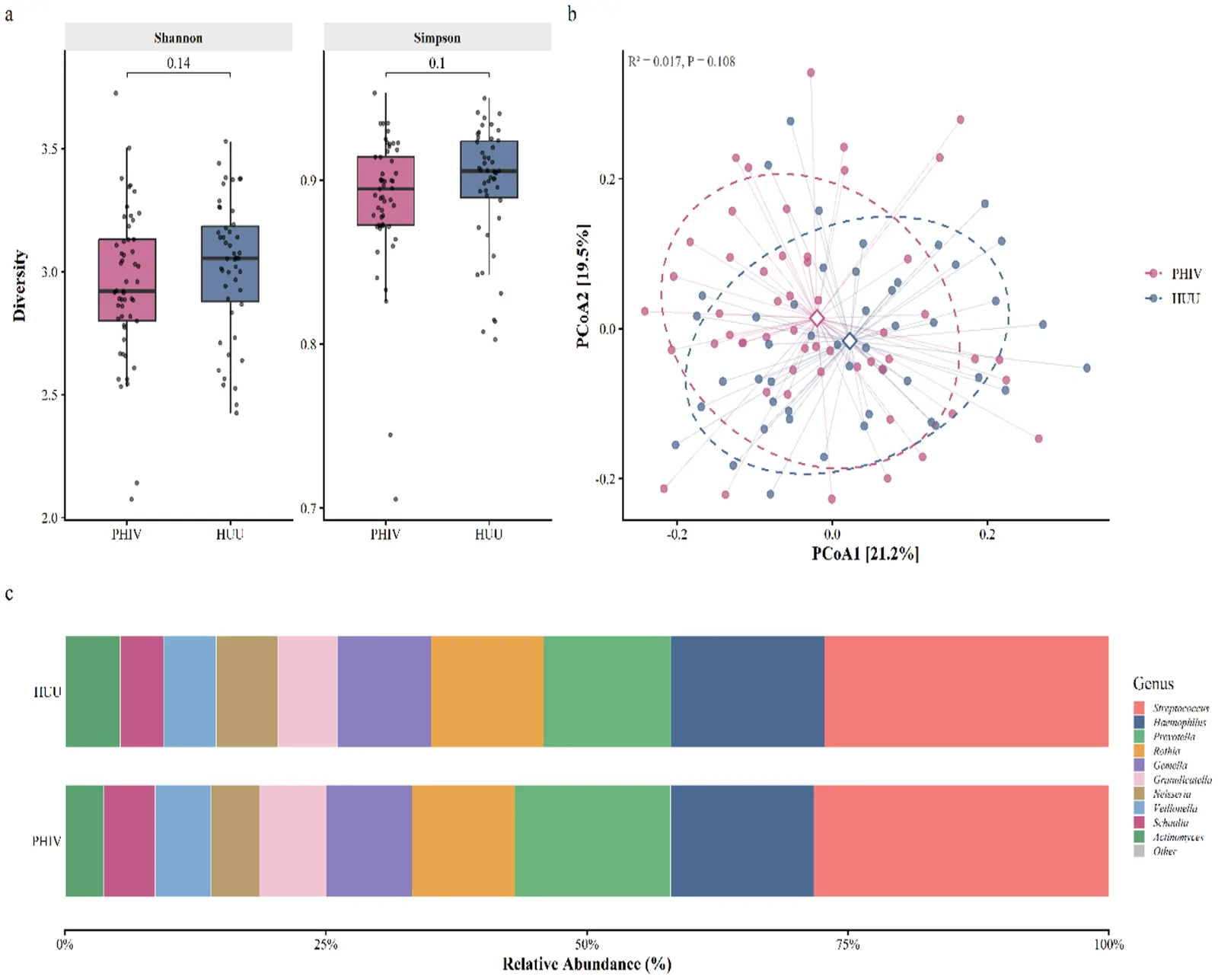
Oral Microbiome Composition and Diversity by HIV Status. a: Alpha Diversity, b: Beta Diversity, C: Relative Abundance

Within-sample diversity did not differ between PHIV and HUU for either the Shannon (p = 0.14) or Simpson (p = 0.10) index (Fig. 1a). Community structure was likewise not significantly different by HIV status on Bray-Curtis PERMANOVA (R^2^=0.017, p = 0.108), with HIV explaining ~ 1.7% of compositional variance (Fig. 1b). Genus-level composition was largely conserved between groups, with *Streptococcus*, *Haemophilus*, *Actinomyces*, and *Rothia* dominant in both strata (Fig. 1c). Because HIV-driven community differences were minimal, all subsequent analyses were stratified by HIV status to evaluate cognition-associated signatures separately within PHIV and HUU.

**Figure 2:**
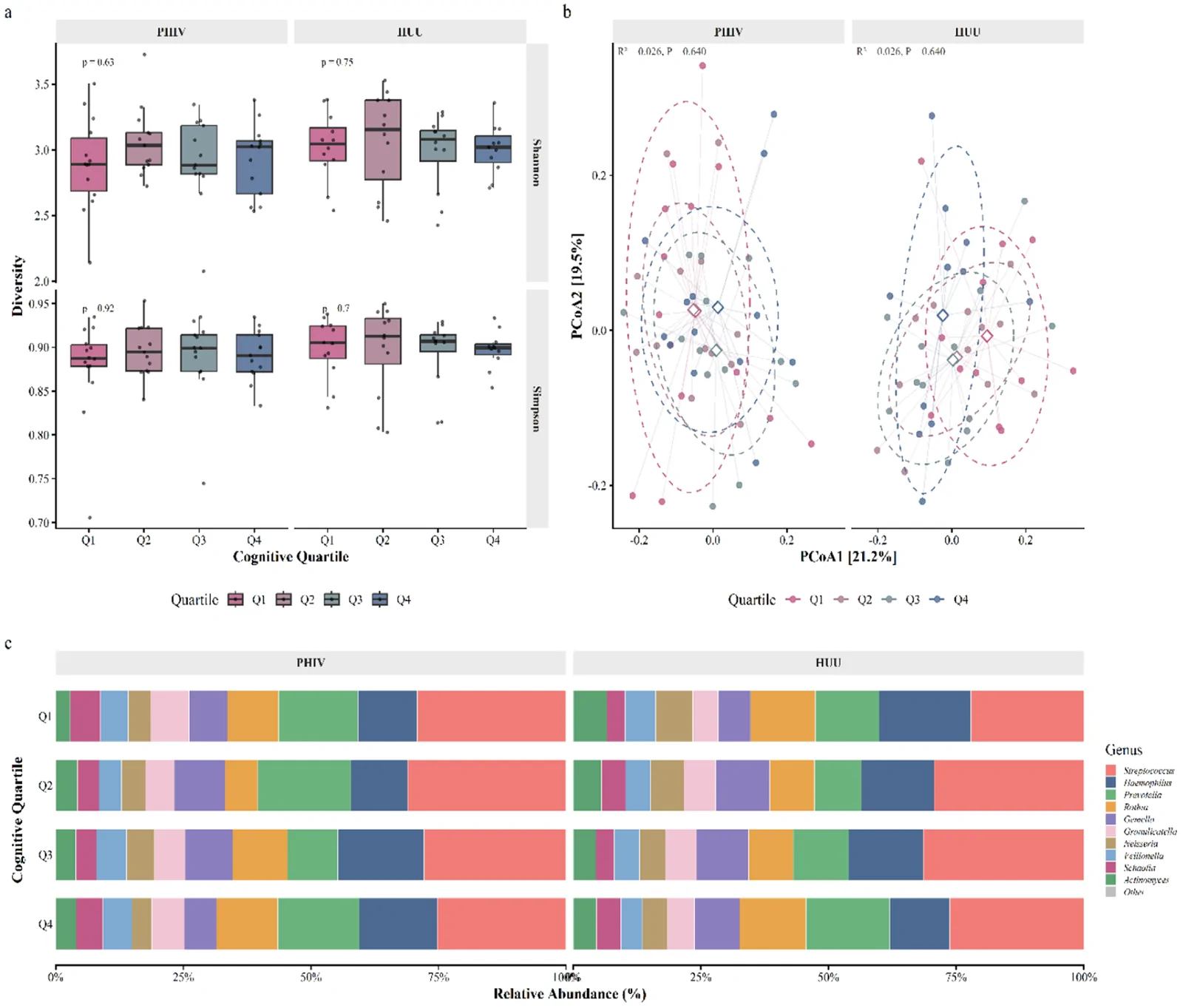
Oral Microbiome By NeuroScreen Scores. a: Alpha Diversity, b: Beta Diversity, C: Relative Abundance

**Figure 3:**
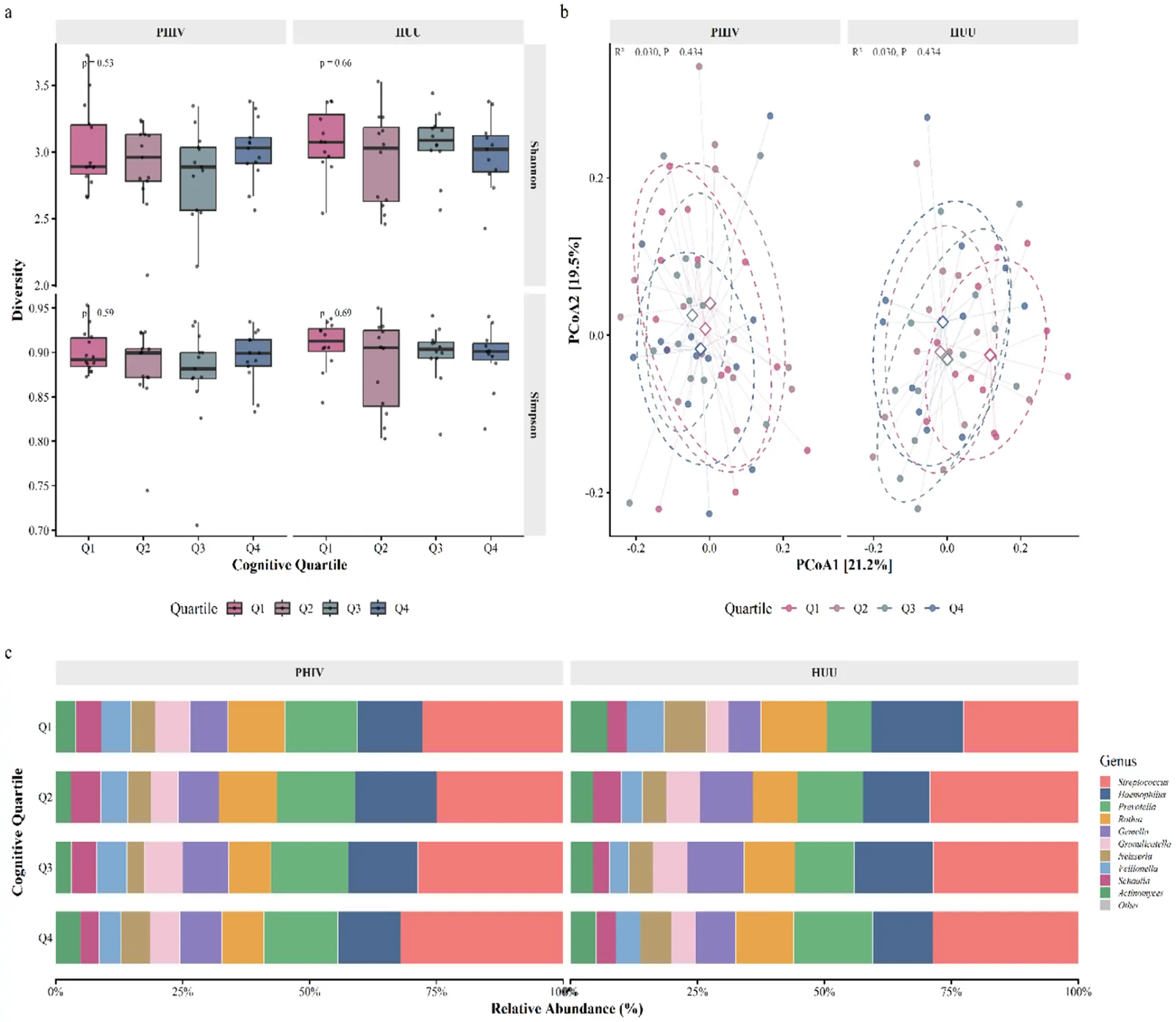
Oral Microbiome By NIH Toolbox Scores. a: Alpha Diversity, b: Beta Diversity, C: Relative Abundance

Neither Shannon nor Simpson diversity was associated with NeuroScreen composite z-score or NIH Toolbox (NIHTB) total cognition score in either HIV stratum (all Kruskal-Wallis p > 0.05 across cognitive quartiles) (Fig. 2a and 3a). Bray-Curtis community structure was also not associated with cognitive scores in either stratum on adjusted PERMANOVA (PHIV NeuroScreen R^2^=0.020, p = 0.37; PHIV NIHTB R^2^=0.013, p = 0.80; HUU NeuroScreen R^2^=0.016, p = 0.71; HUU NIHTB R^2^=0.015, p = 0.75) (Fig. 2b and 3b). The absence of global diversity signals alongside the species-level associations described below is consistent with a compositional, rather than diversity-driven, relationship between the oral microbiome and cognition.

Applying five complementary differential-abundance methods (MaAsLin2^23^, ALDEx2^24^, DESeq2^25^, ANCOMBC2^26^, and radEmu^27^) to continuous cognitive scores, adjusted for age, sex, CD4, CD8, DMFT, OHIS, and gingival inflammation score (GIS), we identified taxa reproducibly associated with cognition within each HIV stratum **(Supplementary Figure S1-5)**. Taxa were considered reproducible when recovered in the same direction by at least two of the five methods among their top-ranked hits at p < 0.05. In PHIV assessed by NeuroScreen, taxa enriched at higher cognitive scores were *Actinomyces israelii* and *Prevotella* species (*P. bivia*, *P. jejuni*), each recovered by two to three methods. Taxa enriched at lower NeuroScreen scores were more numerous and more reproducible, led by *Corynebacterium durum* and *Granulicatella elegans* (four methods each), followed by *Neisseria lactamica*, *Simonsiella muelleri*, and *Streptococcus milleri* (three methods), with additional *Cutibacterium* and *Streptococcus* species recovered by two.

Under the NIHTB in PHIV, the strongest and most reproducible signal was a cluster of taxa enriched at higher cognitive scores and recovered by all five methods: *Tannerella serpentiformis* and *Leptotrichia oral taxa 847* and *498*. These were accompanied by additional *Leptotrichia* species (*L. trevisanii*, *L. hofstadii*, *L. buccalis*, *Leptotrichia sp. HMT-225*) and *Actinomyces israelii*, each recovered by three to four methods. Only *Simonsiella muelleri* was reproducibly enriched at lower NIHTB scores (three methods).

In the HUU stratum under NeuroScreen, the reproducible signal was dominated by taxa enriched at lower cognitive scores: *Corynebacterium matruchotii* was recovered by all five methods, followed by *Kingella denitrificans* and *Neisseria bacilliformis* (four methods each) and several *Aggregatibacter* species and *Simonsiella muelleri* (two to three methods). Taxa enriched at higher NeuroScreen scores in HUU were fewer and less reproducible (*Prevotella nigrescens* and *Lancefieldella sp.*; two methods each). This lower-cognition pattern persisted under the NIHTB in HUU, where *Ottowia sp. oral taxon 894* was recovered by all five methods, alongside *Kingella denitrificans*, *Neisseria bacilliformis*, and *Aggregatibacter actinomycetemcomitans* (four methods each); only *Streptococcus constellatus* and *Prevotella nigrescens* were enriched at higher scores (two methods each).

Across panels, the most reproducible signals were the enrichment of *Leptotrichia* and *Tannerella serpentiformis* at higher cognitive scores in PHIV under the NIHTB, and the enrichment of *Neisseria* and *Kingella* together with *Corynebacterium matruchotii* (NeuroScreen) and *Ottowia oral taxon 894* (NIHTB) at lower cognitive scores in HUU, each recovered by four to five methods. Notably, *Streptococcus* associations differed by stratum: several *Streptococcus* species were enriched at lower cognitive scores in PHIV, whereas *S. constellatus* was enriched at higher scores in HUU.

**Figure 4:**
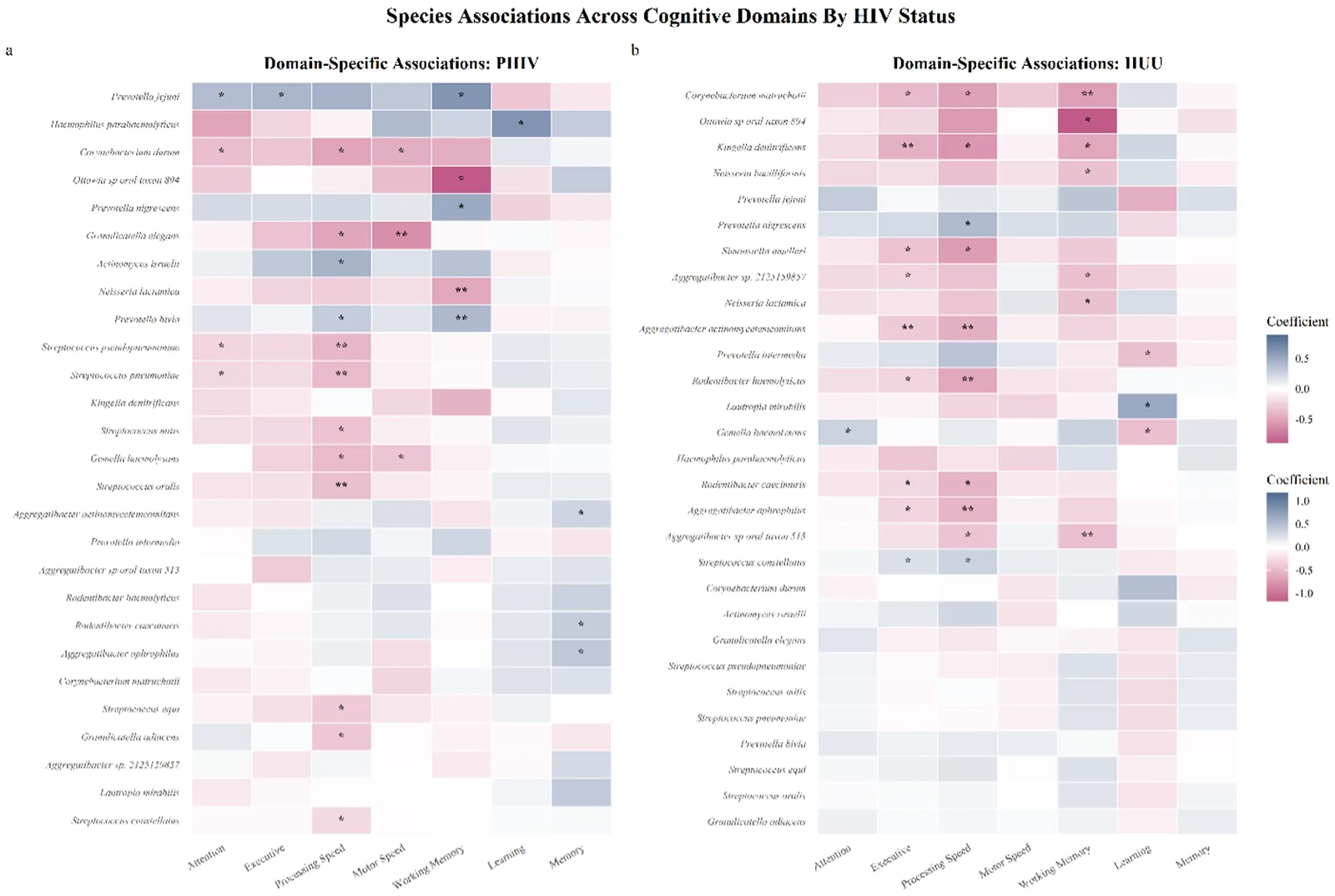
Species Association Across NeuroScreen Cognitive Domains by HIV Status. a: Among PHIV, b: Among HUU

To test whether these associations reflected underlying oral-disease burden, we compared nested MaAsLin2 models with and without adjustment for DMFT, OHIS, and GIS. Effect sizes for consensus taxa were largely stable between minimal and fully adjusted models, indicating that most cognition-associated species retained their associations after controlling for oral health **(Supplementary Figure S6 and S7)**. Because taxon-level effects can emerge without broader community restructuring, we explored overall diversity by collapsing the cognitive-score quartiles into lower- and higher-cognition groups and comparing alpha diversity and beta diversity, stratified by HIV status. Neither alpha nor beta diversity differed significantly between cognitive groups in PHIV or HUU groups **(Supplementary Figure S8 and S9)**, indicating that the cognition-microbiome signal was concentrated at the level of individual taxa rather than reflected in global community structure. In particular, the *Neisseria/Kingella* and *Corynebacterium matruchotii* enrichment at lower cognitive scores in HUU, and the *Leptotrichia/Tannerella serpentiformis* and *Actinomyces* enrichment at higher cognitive scores in PHIV, remained stable across models. To determine whether these associations reflected specific cognitive processes, we tested consensus and abundant taxa against the seven NeuroScreen domain z-scores, stratified by HIV status. In PHIV, domain-level associations concentrated in the processing-speed, motorspeed, and working-memory domains: taxa enriched at lower cognitive scores including *Streptococcus* species (*S. pseudopneumoniae*, *S. pneumoniae*, *S. oralis*, *S. mitis*), *Granulicatella elegans*, and *Gemella haemolysans* associated most strongly with processing speed and motor speed, whereas *Prevotella* species (*P. bivia*, *P. jejuni*) and *Actinomyces israelii*, enriched at higher scores, associated most strongly with working memory and processing speed (Fig. 4a). In HUU, the lower-cognition taxa localized to the executive-function, processing-speed, and working-memory domains, with *Kingella denitrificans*, *Corynebacterium matruchotii*, *Aggregatibacter actinomycetemcomitans*, and *Ottowia oral taxon 894* showing the strongest domain-specific associations (Fig. 4b). These domain-level patterns were nominally significant and hypothesis-generating, suggesting that composite-level signals may be driven by specific cognitive processes rather than distributed uniformly across domains.

## DISCUSSION

To our knowledge, this is the first detailed characterization of oral microbiota signatures in relation to neurocognitive performance in adolescents with or without PHIV. Using shotgun metagenomic sequencing and two validated cognitive instruments treated as continuous outcomes, we found that while global oral microbial diversity and community structure did not significantly differ between adolescents in PHIV and HUU groups, or across cognitive gradients within HIV strata, species-level differential abundance across five complementary methods revealed reproducible taxon-specific associations. The most robust findings were *Neisseria/Kingella* and *Corynebacterium matruchotii* enrichment at lower cognitive scores in HUU, and *Leptotrichia* and *Tannerella serpentiformis* enrichment at higher cognitive scores in PHIV.

The absence of significant global diversity or community structure differences by HIV status is consistent with, and clarified by, prior HIV-oral microbiome studies^28^. Studies in adults have reported increased bacterial richness or altered community composition in PWH compared to uninfected individuals^29^, but these findings have not been consistently replicated across populations, particularly in the pediatric/adolescent age range. In an earlier Nigerian cohort of children aged ≤ 6 years (N = 286) published by our group, HIV infection was only weakly associated with community composition, whereas immune status (low CD4 counts) had a far stronger and more persistent effect^14^. Our PHIV participants were predominantly virally suppressed with relatively preserved CD4 counts (mean 574 cells/μL), a profile that might predict minimal compositional divergence from HUU counterparts. This interpretation also situates our results against Kuhn et al., who found that genus-level compositional shifts in school-age children with HIV on ART in South Africa (notably lower *Neisseria* and *Haemophilus* and higher *Streptococcus*, *Granulicatella*, and *Gemella*), these shifts occurred against a background of only modestly reduced alpha diversity^30^. Beall et al. also concluded that the independent effects of HIV and ART on the oral microbiome, while statistically significant, were modest overall and comparable in magnitude to clinical covariates such as smoking and oral hygiene^31^. Together, these studies suggest that in the era of early, effective ART, it was immunologic control, not necessarily HIV serostatus itself, that governs the pediatric oral microbiome, and that residual signal is best sought at species rather than community-level.

With respect to neurocognition, *Neisseria* and *Kingella* enrichment at lower cognitive scores in HUU was among our most reproducible findings, recovered by four of five methods in both HUU panels, alongside *Corynebacterium matruchotii* (NeuroScreen) and *Ottowia sp. oral taxon 894* (NIHTB), each recovered by all five methods. This direction aligns with emerging species-resolved reports linking *Neisseria* enrichment to poorer cognition in mild cognitive impairment and dementia, contrasting with the genus-level nitrate-reduction literature that more often associates *Neisseria* with better cognition^32-34^. Notably, prior pediatric HIV work found commensal *Neisseria* to be a marker of the healthy oral core that is depleted in dysbiosis^35^, reinforcing that species identity and community context could more strongly determine the cognitive correlate. We therefore caution against extrapolating from genus-level nitrate biology as it is very likely that genus-level generalizations obscure species-specific effects^36^.

In PHIV, better NeuroScreen performance was associated with *Actinomyces israelii* and *Prevotella* species (*P. bivia*, *P. jejuni*), recovered by two to three methods. *Actinomyces* are gram-positive commensals linked to favorable immune profiles^37^ and *Prevotella* associations, from a genus containing both commensal and pathogenic members, warrant more caution^38,39^. Under the NIHTB, the strongest signal in better performers was a cluster recovered by all five methods *Tannerella serpentiformis* and *Leptotrichia oral taxa 847* and *498* accompanied by additional *Leptotrichia* species and *A. israelii*. Notably, *Leptotrichia*-cognition associations are direction-dependent: higher subgingival *Leptotrichia* has been linked to worse cognition in adults via LPS-mediated neuroinflammation^40^, yet commensal *Leptotrichia* was depleted in post-stroke cognitive impairment and in centenarians with dementia^41-43^. The consistency of this *Leptotrichia/Tannerella* signal across all five methods is most parsimoniously interpreted as a marker of biofilm ecological maturity reflecting an underlying oral ecological state, not necessarily a direct neuroactive effect. This finding suggests a depletion-with-impairment pattern in the commensal literature and should be tested directly through functional pathway profiling in longitudinal follow-up.

A key strength of our analytic framework was the ability to distinguish cognition-associated taxa from oral-disease-associated taxa. Given emerging evidence from our group and others, linking oral health (DMFT) to poorer cognition in similar populations^44^, the possibility that microbial signals simply mark underlying oral disease is a legitimate concern. Our nested-model sensitivity analysis directly addressed this by comparing minimal and fully oral-health-adjusted models (adjustments for oral health measures such as DMFT, OHI-S, and gingival inflammation). We observed stability of effect sizes for *Neisseria/Kingella*, *Corynebacterium matruchotii*, and the PHIV *Leptotrichia/Tannerella* signals across models suggesting that these associations are not likely to be mere downstream markers of oral disease and may reflect cognition-relevant microbial biology operating at least partly independently of the caries and periodontal-inflammation pathways that conventionally link the mouth to the brain.

Domain-specific analyses further localized the HUU lower-cognition signal to the executive-function, processing-speed, and working-memory domains, and the PHIV signals to the processing-speed, motorspeed, and working-memory domains, suggesting taxon-specific pathways with domain-selective effects. Notably, *Streptococcus* associations were direction-discordant by HIV stratum with several *mitis*- and *suis*-group species being enriched at lower cognitive scores in PHIV, while *S. constellatus* was enriched at higher scores in HUU, underscoring that the same genus may carry stratum-dependent meaning.

Several non-mutually exclusive mechanistic pathways may underline these associations linking oral microbial taxa to cognition in this age group. Chronic oral inflammation can drive systemic elevations in IL-1β, IL-6, and TNF-α that compromise blood-brain-barrier integrity and prime microglia, a cascade characterized in the periodontitis-to-neuroinflammation literature^45^. Periodontal pathogens and their products may also reach the CNS through hematogenous spread, LPS-mediated barrier disruption, and immune-cell trafficking^46^. In parallel, salivary translocation^47,48^ to the gut can reshape enteric communities and downstream immune and metabolic signaling along an oral-gut-brain axis, with shortchain fatty acid production and modulation of systemic nitric oxide bioavailability via the nitrate-nitrite-NO pathway, the latter directly relevant to the *Leptotrichia* finding^32,49,50^. The reproducibility of the *Neisseria/Kingella* signal across HIV strata suggests some cognition-associated patterns are not HIV-specific. In PHIV, however, chronic immune activation and cumulative ART exposure may lower the threshold at which oral dysbiosis translates into detectable cognitive effects via the aforementioned mechanisms^51,52^. As these mechanisms are largely derived from adult and animal models, their relevance to virally suppressed adolescents remain to be established and is offered as a framework for future functional follow-up studies.

A further consideration is the heterogeneity of the two cognitive instruments. NeuroScreen and the NIH Toolbox Cognition Battery capture partly distinct constructs - NeuroScreen is weighted toward timed processing and motor speed, whereas the NIH Toolbox Total Cognition composite incorporates crystallized abilities (vocabulary and oral reading) that are strongly influenced by language exposure and educational opportunity. Consistent with this, the largest HIV-specific differences in our cohort were observed on the crystallized NIH Toolbox subtests, and the two instruments recovered largely non-overlapping sets of consensus taxa within the same participants. This divergence is expected given that i) HIV-associated cognitive effects are domain-specific, not uniform across all domains^53^, ii) composite domain scores can be more sensitive than global aggregates in this population^54^, and iii) the NIH Toolbox was normed on US English-speaking populations with validation in adolescents in SSA still emerging^55,56^. This heterogeneity argues for tool-specific interpretation but at the same time, we highlight the taxa recovered concordantly across both instruments (i.e., notably *Neisseria/Kingella* signal in HUU adolescents) warranting greater confidence in our findings.

Strengths include the focus on PHIV in SSA, the population most affected yet least studied; species-level metagenomic resolution; two validated instruments enabling cross-instrument comparison; and five complementary differential-abundance methods. The main limitation of our study is the modest sample size (N = 100), which may have limited power to detect smaller effects after multiple-testing correction, compounded by imbalance in the NIHTB classification (only 22 of 100, and 5 of 53 PHIV, classified above average). The cross-sectional design precludes causal inference and directionality as dysbiosis may precede cognitive impairment, or cognitive impairment may drive behavioral changes altering the microbiome. The detection of *Streptococcus suis*, a porcine pathogen not native to the human oral cavity, in many samples suggests that some species-level assignments may reflect misclassification of reads from closely related oral streptococci or low-level contamination, warranting cautious interpretation of *Streptococcus* findings. Finally, unmeasured confounders such as diet, host genetic factors, and periodontal health likely influence the oral microbiome despite adjustment.

Future work should include longitudinal follow-up to test whether microbiome trajectories predict cognitive change, functional pathway analysis to clarify SCFA and NO involvement, and replication in other SSA populations. If a causal role is supported, oral microbiome-targeted interventions will warrant further evaluation for improving cognitive outcomes in this population.

In conclusion, the oral microbiome harbors distinct taxon-level signatures associated with neurocognitive performance among adolescents with and without HIV in SSA. Importantly, these associations differed by HIV status, suggesting that HIV may modify the relationship between oral microbial ecology and neurocognitive function even in the absence of marked differences in overall community structure. Together, these findings position the oral microbiome as an accessible window into biological processes relevant to neurocognitive health and support its potential utility for biomarker discovery and risk stratification. Longitudinal and mechanistic studies are now needed to determine whether these microbial signatures precede neurocognitive changes and represent modifiable targets for improving neurocognitive outcomes in adolescents living with HIV. Together, these findings identify the oral microbiome as a potential biomarker of neurocognitive health and a candidate target in this population, motivating future longitudinal, mechanistic, and interventional studies.

## METHODS

### Study Participants

This cross-sectional analysis used baseline data from 53 PHIV and 47 HUU adolescents aged 10–13 years enrolled in the uBLOoM (*Uncovering the Biological Link between Oral Microbiota and Mental Health in Adolescents Living with HIV*) observational cohort.^57,58^ Participants were recruited at the University of Benin Teaching Hospital (UBTH), Nigeria. PHIV were recruited from the UBTH’s Special Treatment Clinic and HUU from the Department of Family Medicine. Groups were matched by age (± 0.5 years), sex, and socioeconomic status. Eligibility criteria required PHIV to have documented perinatal HIV acquisition and current ART use. Participants were excluded if they were currently enrolled in another clinical study that could interfere with study procedures or had any condition that would prevent full compliance with or completion of study activities. Individuals who indicated interest and met the eligibility criteria were enrolled in the cohort. The study was conducted in accordance with the ethical principles of the Declaration of Helsinki and its subsequent amendments. The protocol was reviewed and approved by Institutional Review Boards (IRBs) at Rutgers State University of New Jersey (protocol [Pro2022000949]) and the UBTH, Benin City (ADM/E22/A/VOL. VII/148301112). Written informed consent was obtained from a parent of legal guardian for each minor participant, and written assent was obtained from each youth before recruitment. Additional verbal consent was obtained from participants for oral sample collection. Research staff provided standardized written and verbal explanations about the study procedure prior to their enrollment, ensuring their voluntary participation, comprehension of potential risks and benefits, and right to withdraw from the study at any time without penalty.

### Demographics, Clinical, and Oral Health Measures

Trained staff administered structured questionnaires capturing demographics (age, sex, education), medical and behavioral history (antibiotic use, prenatal and perinatal history, and substance use). For PHIV, HIV-specific data (age at diagnosis, ART regimen and duration, most recent CD4 + and CD8 + count, and viral load) were extracted from medical records. Oral health was assessed by self-reported questionnaires and standardized clinical examinations including, but not limited to hygiene practices, DMFT, OHI-S, and GIS.

#### Neurocognitive Assessment:

Cognitive function was evaluated using two validated instruments: NeuroScreen^59^ (Version 4.4.9), validated for use in SSA and, NIHTB Cognition Battery^60^ (V3 3.10.1), a standardized assessment of multiple cognitive domains.

NeuroScreen spans six neuropsychological domains through ten rapid screening tasks, providing a global neurocognitive impairment score. For this study, we followed the approach described by Robbins et al. (2018)^61^. All raw NeuroScreen subtest scores were standardized to z-scores across the study cohort. Timed subtests (Trail Making 1, 2, and 3 completion times, and Number Speed total time) were reverse coded so that higher values reflected better cognitive performance. A composite z-score was generated for each participant by averaging the standardized z-scores across the twelve available subtests (participants with fewer than six valid subtests were excluded). In parallel, we derived domain-specific z-scores by averaging subtest z-scores within each of seven cognitive domains: attention, executive function, processing speed, motor speed, working memory, learning, and memory.

NIHTB Cognition Battery performance was summarized using the age-adjusted standard score for the Total Cognition Composite, along with the seven individual domain-specific tests as previously described.

Both the NeuroScreen composite z-score and the NIHTB Total Cognition Composite were treated as continuous outcomes in all primary microbiome analyses to preserve statistical power and avoid arbitrary threshold effects. In sensitivity analyses, we additionally categorized cognitive performance rather than modeling it continuously: NeuroScreen composite scores were grouped into quartiles based on their percentile distribution within the cohort, and NIHTB Total Cognition performance was categorized using the age-adjusted standard scores. These categorical groupings were used to corroborate the continuous outcome findings and are reported in the **Supplementary File**.

### Oral Microbiome Sample Collection and Processing

Oral rinse samples were collected via standardized saline gargle protocol^62^ where participants rinsed with 10 mL sterile normal saline (0.9% NaCl) for 15 seconds and gargled with their heads tilted back for an additional 15 seconds. The expectorated sample was collected in sterile 15-mL conical tubes and immediately stored at 4°C. Samples were processed within 6 hours of collection by centrifugation at 2,000×g for 15 minutes at 4°C. The supernatant was removed and the microbial pellet resuspended in ~ 4,500 μL saline, then aliquoted into cryovials and stored at −80°C until DNA extraction.

### Metagenomic Sequencing and Bioinformatic Analysis

Microbial DNA was extracted using the DNeasy PowerSoil Pro Kit^63^ (Qiagen) per manufacturer's protocol with an additional bead-beating step (2 × 1 min at 6.0 m/s) to enhance lysis of Gram-positive organisms. Yield and purity were assessed by fluorometry (Qubit 4.0, Thermo Fisher Scientific) and spectrophotometry (NanoDrop One). Samples with DNA yield < 1 ng/μL or A260/280 outside the range of 1.7–1.9 underwent a second extraction attempt; samples failing quality thresholds were excluded from downstream analysis. Shotgun metagenomic sequencing libraries were prepared using the Nextera XT DNA Library Preparation Kit (Illumina)^64^ with input DNA normalized to 1 ng. Sequencing was performed on the Illumina NovaSeq 6000 platform (150-bp paired-end reads; ~20 million read-pairs per sample) as previously described^65^. Low-quality reads and those mapped to human DNA were removed as in the previous work. The processed data were deposited in the NCBI Sequence Read Archive under BioProject PRJNA1345363. We then assigned species-level taxonomy for quality-filtered, host-depleted reads using Kaiju (v1.10.1)^66^ by aligning them to completely assembled and annotated reference genomes of archaea, bacteria, and viruses from the NCBI RefSeq database. The resulting species count/abundance data and metadata were imported and managed as *phyloseq* objects in R (version 4.5.1).

### Taxon filtering

Differential abundance testing was restricted to prevalent taxa to limit zero inflation. Species were agglomerated to the species rank and retained only if present at ≥ 3 reads in ≥ 25% of samples with mean relative abundance ≥ 1×10^−4^ across samples. This filter was applied identically across all the methods so that between-method comparisons reflected differences in statistical model rather than differences in the feature set tested.

### Statistical Analysis

Per-sample species-level counts were assembled into a single feature table with full seven-rank lineages parsed from Kaiju output and stored as a *phyloseq* object. Alpha diversity was assessed using Shannon and Simpson indices and compared across HIV groups by Wilcoxon rank-sum tests and across cognitive quartiles by Kruskal-Wallis tests. Beta diversity was assessed using Bray-Curtis dissimilarity on within-sample relative abundances with statistical testing by PERMANOVA. Genus-level relative abundance was visualized as stacked bars showing the top 10 most abundant genera across groups.

Species-level differential abundance was evaluated by treating cognition as a continuous predictor using MaAsLin2^23^, ALDEx2^24^, DESeq2^25^, ANCOMBC2^26^, and radEmu^27^ approaches. This multi-method design leverages methodological diversity across compositional (MaAsLin2, ALDEx2), count-based (DESeq2), bias-corrected compositional (ANCOMBC2), and reference-taxon-free (radEmu) frameworks to identify robust taxon-cognition associations. Analyses were stratified by HIV status and conducted separately for each continuous cognitive outcome, yielding four analytic panels per method. All models were adjusted for age, sex, CD4 and CD8 counts, dental caries burden (DMFT), OHIS, and GIS. Effect sizes represent the change in species abundance (in method-specific units) per one-unit increase in cognitive score; positive coefficients therefore indicate species enriched at higher cognitive scores. Taxa were considered cross-method consistent when identified in the same direction by at least two of the five methods in a given panel.

To evaluate whether cognition-associated taxa represented direct associations or reflected underlying oral disease burden, we conducted a sensitivity analysis for each consensus taxon by fitting two nested MaAsLin2 models: a minimal model adjusted for age, sex, CD4, and CD8 only, and a fully adjusted model additionally including DMFT, OHIS, and GIS. Coefficients from both models were compared; stable coefficients between models indicated that a taxon's association with cognition was independent of oral health, whereas substantial attenuation (> 30% shrinkage in effect size) suggested oral disease as a partial explanation of the association.

To identify whether cognition-associated taxa related preferentially to specific cognitive processes rather than to global cognition, we ran MaAsLin2 separately for each of the seven NeuroScreen domain z-scores as continuous outcomes, stratified by HIV status, with the full covariate adjustment. Results were visualized as a species-by-domain heatmap displaying model coefficients with significance annotation.

## Supplementary Material

This is a list of supplementary files associated with this preprint. Click to download.

• SupplementaryMaterials.docx

## Figures and Tables

**Table 1:** Baseline characteristics of the uBLOoM cohort stratified by HIV status.

Characteristics	PHIV (N = 53)	HUU (N = 47)	Total (N = 100)	p-value
Sociodemographic				
Age (Years), Mean (SD)	10.47 (0.77)	10.28 (0.99)	10.38 (0.89)	0.20
Gender, N (%)	20 (38)	20 (43)	40 (40)	0.68
Males				
Delivery Method, N (%)	47 (89)	33 (70)	80 (80)	0.04
1. Vaginal	3 (5.7)	3 (6.4)	6 (6)	
2. C-Section	3 (5.7)	7 (15)	10 (10)	
3. Assisted Vaginal	-	4 (8.5)	4 (4)	
4. Emergency C-Section				
Weight (Kg), Mean (SD)	29. 3 (6)	31 (6.5)	30.1 (6.3)	0.12
Height (cm), Mean (SD)	140 (9)	141 (9)	141 (9)	0.65
BMI (kg/m^2^), Mean (SD)	14.75 (1.98)	15.39 (2.13)	15.05 (2.06)	0.07
Education, N (%)	39 (74)	29 (62)	68 (68)	0.28
1. Primary	14 (26)	18 (38)	32 (32)	
2. JSS				
Alcohol Use Ever, N (%)	4 (7.5)	5 (11)	9 (9)	0.73
Yes				
Alcohol Use Frequency, N (%)	1 (25)	-	1 (1)	0.44
1. At least once a week	3 (75)	5 (100)	8 (89)	
2. At least once a month				
Oral and Dental Health				
DMFT Score, Mean (SD)	0.55 (1.15)	0.32 (0.91)	0.44 (1.05)	0.17
PUFA Score, Mean (SD)	0.03 (0.19)	0.04 (0.2)	0.04 (0.19)	0.9
OHIS Category, N (%)	20 (38)	17 (36)	37 (37)	> 0.99
1. 0-1.2: Good	29 (55)	27 (57)	56 (56)	
2. 1.3-3: Fair	4 (7.5)	3 (6.4)	7 (7)	
3. >3.1: Poor				
Gingival Index Score Category, N (%)	22 (42)	18 (38)	40 (40)	0.68
1. 0: None	31 (58)	28 (60)	59 (59)	
2. 0.1-1: Mild	-	1 (2.1)	1 (1)	
3. 1.1-2: Moderate				
Clinical Features				
CD4 Count, Mean (SD)	574 (219)	934 (312)	743 (321)	< 0.001
CD8 Count, Mean (SD)	745 (483)	387 (118)	577 (402)	< 0.001
Viral Load, N (%)	15 (28)	-	15 (15)	
1. Detectable ( > = 20 copies)	38 (72)	-	38 (38)	
2. Not Detectable (< 20 copies)				

**Table 2:** Neurocognitive Scores of the uBLOoM cohort stratified by HIV status.

Characteristics	PHIV (N = 53)	HUU (N = 47)	Total (N = 100)	p- value
NeuroScreen Scores				
Completion Time, Mean (SD)	39.5 (18.04)	29.51 (34.43)	34.72 (21.75)	0.02
Finger Tapping (Dominant), Mean (SD)	215.91 (28)	208.98 (33.24)	212.65 (30.62)	0.26
Finger Tapping (Non-Dominant), Mean (SD)	189.21 (23.26)	188.11 (26.28)	188.69 (24.61)	0.82
Number Span Backward, Mean (SD)	1.23 (1.19)	1.17 (1.32)	1.2 (1.25)	0.82
Number Span Forward, Mean (SD)	3.62 (1.08)	3.85 (1.06)	3.73 (1.07)	0.28
Number Speed, Mean (SD)	51.31 (14.5)	47.67 (13.69)	49.6 (14.17)	0.19
Trail Making Test 1, Mean (SD)	43.36 (20.01)	36.54 (17.27)	40.15 (18.99)	0.07
Trail Making Test 2, Mean (SD)	50.62 (22.5)	43.21 (20.23)	47.14 (21.68)	0.08
Trail Making Test 3, Mean (SD)	23.87 (16.58)	20.42 (10.65)	22.25 (14.14)	0.21
Verbal Learning Delayed Recall, Mean (SD)	4.19 (1.53)	4.53 (1.41)	4.35 (1.48)	0.24
Verbal Learning Total, Mean (SD)	8.91 (1.68)	9.17 (0.94)	9.03 (1.38)	0.32
Visual Discrimination 1, Mean (SD)	11.11 (4.56)	11.7 (3.87)	11.39 (4.24)	0.48
Visual Discrimination 2, Mean (SD)	19.49 (4.87)	21.72 (4.92)	20.54 (4.99)	0.02
NIH Toolbox Scores				
Oral Reading, Mean (SD)	65 (28)	89 (36)	76 (34)	< 0.001
Picture Vocabulary, Mean (SD)	50 (15)	62 (18)	56 (17)	< 0.001
Dimensional Card Sort, Mean (SD)	86 (11)	92 (13)	89 (12)	0.02
Flanker, Mean (SD)	72 (14)	80 (14)	76 (14)	0.01
Working Memory, Mean (SD)	88 (13)	95 (15)	91 (15)	0.01
Processing Speed, Mean (SD)	89 (11)	93 (13)	91 (12)	0.07
Picture Sequence Memory, Mean (SD)	87 (13)	94 (11)	90 (13)	0.02
Crystalized Composite, Mean (SD)	55 (20)	77 (30)	65 (28)	< 0.001
Fluid Composite, Mean (SD)	77 (14)	87 (14)	82 (15)	0.001
Total Cognition, Mean (SD)	59 (18)	78 (23)	68 (22)	< 0.001

## Data Availability

The metagenomic sequence data generated in this study are available in the NCBI Sequence Read Archive under BioProject accession PRJNA1345363. Processed species-count tables, participant metadata (de-identified), and analysis code are available from the corresponding author on reasonable request.
